# EDOF intraocular lens design: shift in image plane vs object vergence

**DOI:** 10.1186/s12886-023-03144-4

**Published:** 2023-10-02

**Authors:** Pooria Omidi, Alan Cayless, Achim Langenbucher

**Affiliations:** 1 Department of Experimental Ophthalmology, Saarland University, 66424 Homburg, Saarland Germany; 2 School of Physical Sciences, The Open University, MK7 6AA Milton Keynes, UK

**Keywords:** Cataract surgery, Intraocular lens, Ray tracing

## Abstract

**Background:**

To compare 2 different design scenarios of EDOF-IOLs inserted in the Liou-Brennan schematic model eye using raytracing simulation as a function of pupil size.

**Methods:**

Two EDOF IOL designs were created and optimized for the Liou-Brennan schematic model eye using Zemax ray tracing software. Each lens was optimized to achieve a maximum Strehl ratio for intermediate and far vision. In the first scenario, the object was located at infinity (O1), and the image plane was positioned at far focus (I1) and intermediate focus (I2) to emulate far and intermediate distance vision, respectively. In the second scenario, the image plane was fixed at I1 according to the first scenario. The object plane was set to infinity (O1) for far-distance vision and then shifted closer to the eye (O2) to reproduce the corresponding intermediate vision. The performance of both IOLs was simulated for the following 3 test conditions as a function of pupil size: a) O1 to I1, b) O1 to I2, and c) O2 to I1. To evaluate the imaging performance, we used the Strehl ratio, the root-mean-square (rms) of the spot radius, and the spherical aberration of the wavefront for various pupil sizes.

**Results:**

Evaluating the imaging performance of the IOLs shows that the imaging performance of the IOLs is essentially identical for object/image at O1/I1. Designed IOLs perform dissimilarly to each other in near-vision scenarios, and the simulations confirm that there is a slight difference in their optical performance.

**Conclusion:**

Our simulation study recommends considering the difference between object shift and image plane shift in design and test conditions to achieve more accurate pseudoaccommodation after cataract surgery.

**Supplementary Information:**

The online version contains supplementary material available at 10.1186/s12886-023-03144-4.

## Background

In recent decades, ophthalmic surgeons have had the choice of alternative intraocular lens (IOL) designs in addition to classical spherical lenses. Besides toric lenses for correction of corneal astigmatism, refractive and diffractive bifocal or multifocal lenses (MF), enhanced depth of focus lenses (EDOF), and monofocal plus lenses have been developed to maintain pseudoaccommodation after cataract surgery. Moreover, with cataract surgery, the surgeon may aim for postoperative monovision, in which the dominant eye is corrected for far distance objects and the non-dominant eye is corrected for some amount of near vision, typically for objects at distances between 0.5 to 1.5 m [1, 2]. MF lenses generate images simultaneously from objects positioned at varying distances from the retina [3]. These IOLs usually have a high near addition for activities that require near and intermediate vision, such as reading and working with tablets and mobile phones. Unlike multifocal IOLs used in the treatment of presbyopia, EDOF lenses employ distinct strategies to uphold clear vision for both far and intermediate distances. Some EDOF IOLs stretch the focal point to encompass near additions within a broad focal range. These lenses do not exhibit dedicated far, intermediate, or near focuses. Instead, they provide continuous vision for objects situated at far to intermediate distances, with a plateau in the corresponding defocus curve. Conversely, another group of EDOF IOLs presents a bifocal power profile designed for specific wavelengths and pupil sizes. The diffractive profile of these IOLs exhibits distinct efficiency levels for each wavelength. By combining foci with varying efficiencies, these lenses create an extended focal point for enhanced light distribution [4, 5].

Although both multifocal and EDOF lenses promise to increase levels of spectacle independence, they may cause unwanted photic effects such as glare and halos [6]. Generally, the aim of presbyopia-correcting IOLs is to focus an image at the retina of objects located at different distances and they, therefore, enhance defocus at near and intermediate distances by changing the optical power of the eye [7]. In theory, changes in the optical power of the pseudophakic eye can be achieved by axial displacement or changes in lens thickness or curvature accordingly (If a fixed refractive index for the IOLs is assumed). Basically, monofocal IOLs are designed and optimized for the best visual acuity for distant objects imaged on the retina. In contrast, MF and EDOF lenses seem to be optimized for objects at infinity to achieve the best imaging performance at different focal planes. Unfortunately, this approach is not realistic since corneal and lens aberrations change with various object distances [8, 9]. Consequently, to simulate under more realistic conditions such MF or EDOF lenses have to be designed and evaluated with objects at varying distances and a fixed retinal position. The main objective of this study was to show the effect of 2 design strategies on the imaging performance of EDOF IOLs. Whether the object is located at infinity and the focal planes change (common design and test situation) or the focal plane is fixed at the retinal position, the object vergence changes (realistic but uncommon design and test situation). The 2 lens designs were compared for image performance for far vision and for near vision achieved by a shift in the object plane or a shift in the image plane. All simulations were performed for various pupil sizes to extract the Strehl ratio, root-mean-square (rms) of the spot radius, and primary spherical aberration of the wavefront error.

## Methods

### Eye model specifications

A pseudophakic model eye was implemented for this simulation study. The model eye used for optical simulations is based on the Liou-Brennan schematic model eye introduced in 1997 [10]. The Liou-Brennan model eye is characterized by four coaxial refracting surfaces and has an equivalent power of 60.35 D and an axial length of 23.95 mm. The cornea of this model is defined by two rotationally symmetric surfaces with a thickness of 0.5 mm. These two rotationally symmetric surfaces (front and back surfaces) are described by radii of 7.77 and 6.40 mm, and asphericities of -0.18 and -0.60, respectively. The pupil is slightly decentered by 0.5 mm in the nasal direction with respect to the optical axis to consider an incident ray angle of $5^{\circ }$ from the nasal direction such that an incident ray bundle passes through the aperture stop and is focused on the fovea. The fovea is located about 1.4 mm temporally from the optical axis. To build the pseudophakic eye model, the natural lens of the model eye is initially removed from this model. The IOL is placed within the model eye so that the equator of the IOL aligns with the equatorial position of the natural lens. Consequently, the IOL is positioned 1 mm behind the pupil along the optical axis of the model eye. Table 1 lists all the relevant parameters of the proposed phakic and pseudophakic model eye. Note that the IOL in this table is a basic monofocal aspheric IOL with an equivalent power of 20 D and the front surface of the IOL reforms after optimizing it to extend the depth of focus.

**Table 1:** Structural parameters of the phakic and pseudophakic eye model. (The unrepeated pseudophakic surface parameters are denoted by an Asterisk* symbol)

	Medium	Radius [mm]	Asphericity	Thickness [mm]	Optical diameter [mm]
Surface					
1	Cornea	7.77	-0.18	0.5	14
2	Aqueous	6.40	-0.6	3.16	14
3	Pupil	$\infty$	-	0	0.5 to 6
4	Crystalline Lens	12.40	-0.94	4.02	10
5	Vitreous	-8.10	0.96	16.27	10
6	Retina	-12	-	-	24
Surface*					
3*	Aqueous	$\infty$	-	1	0.5 to 6
4*	Hydrophilic IOL	15.79	-1.5	0.718	6
5*	Vitreous	-15.79	-	18.572	6

### EDOF design and optimization

In this study, ray tracing was conducted using the ZEMAX Professional ray tracing software (Version 19.8, Washington, USA) in the sequential ray tracing mode. The simulations employed a monochromatic light source with a wavelength of 500 nm.

To convert an aspheric lens into an EDOF lens and alter the beam path, we employed more complex geometries rather than the traditional rotationally symmetric surfaces. These refractive surfaces differ significantly from spherical and aspheric designs, enabling them to address optical aberrations and adjust the lens’s power profile. In commercial EDOF IOLs, the anterior surface is typically engineered using rotationally symmetric functions, such as circular Zernike polynomials, with coefficients tailored to the spherical aberration family.

To design EDOF IOLs, first, we divided the front surface into three different concentric annular zones. The purpose is to assign different optimization criteria for each zone and apply a weighting function to the entire surface accordingly. In this study, the Strehl ratio is employed as the optimization criterion. The Strehl ratio, which quantifies the average deviation of the wavefront from its ideal shape, is calculated using the root-mean-square (rms) wavefront error using the following formula.

1 $$\begin{aligned} S=e{\frac{-4\pi ^2\sigma ^2}{\lambda ^2}} \end{aligned}$$

The annular zones are labelled as small (0 to 1.5 mm), medium (1.5 to 3 mm), and large (3 to 6 mm). Each of these zones is optimized for particular vision distances. The small and large zones are optimized to maximize the Strehl ratio for intermediate and far vision respectively. Therefore, the central region of the IOLs has the highest refractive power (intermediate vision) and the peripheral region has the lowest (far vision). The medium zone is optimized to achieve the highest possible Strehl ratio for far and intermediate vision simultaneously. The main function of the medium zone is to act as an interface between the small and large zones and to produce a continuous refractive power over the entire surface, maintaining the refractive power transition to be as smooth as possible.

It’s important to note that each zone cannot operate entirely independently, and optimizing one zone may have a slight influence on others. As intermediate and far vision are not equally utilized by the patient, a weighting function is applied in the optimization procedure. Specifically, 50% of the weighting function is assigned to far vision (large zone), while 40% is allocated to intermediate vision (small zone). The medium zone receives a 10% allocation, shared between far and intermediate vision. Optical designers can adjust these weighting functions as needed to optimize each zone with different contributions to the final image quality.

### Design scenarios and test conditions

**Fig. 1:**
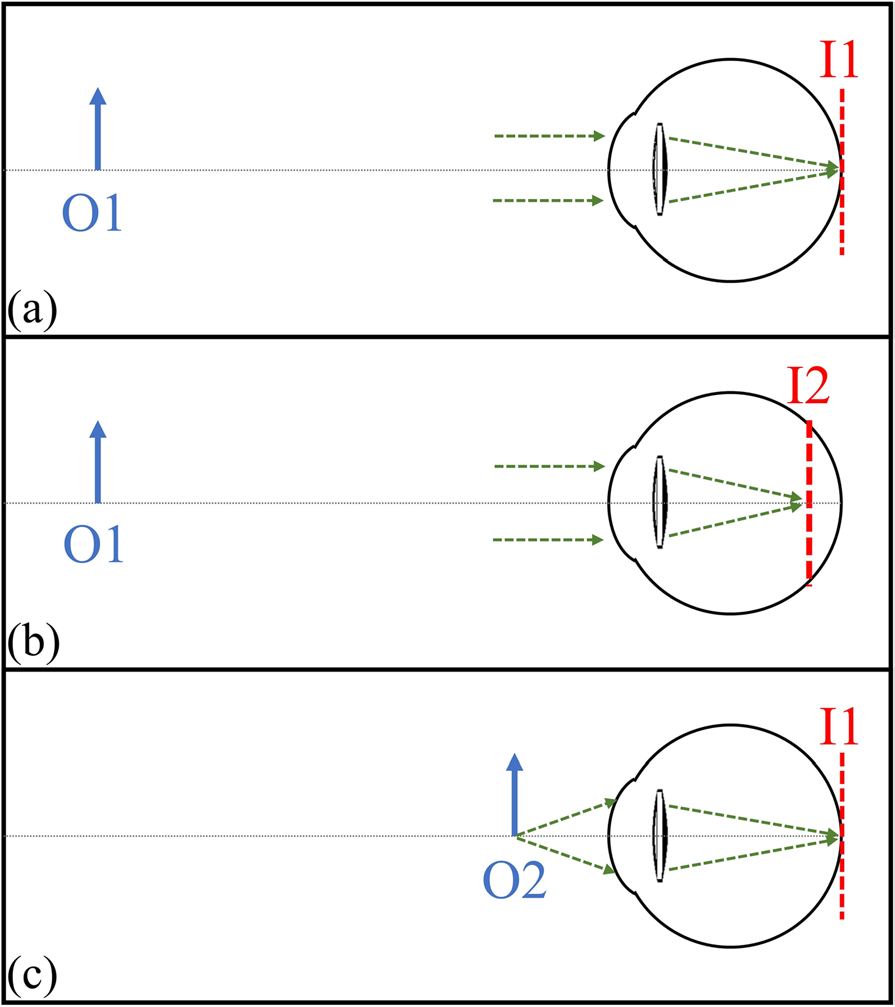
Schematic illustration of the object and image planes in different conditions. **a** Far vision. **b** Intermediate vision in scenario 1. **c** Intermediate vision in scenario 2

Far vision is modelled under a single condition in which the object vergence is set to infinity (O1), and the image plane is positioned at the original Liou-Brennan eye image plane (I1). To emulate the intermediate vision condition, two different scenarios are applied. In the first scenario, which is more common, the image plane is shifted forward on the optical axis (I2) while the object remains located at infinity (O1). In our model, the image plane is shifted about 0.28 mm into the vitreous chamber to add 1.5 D to the center of the lens. However, this scenario does not accurately represent the intermediate vision of the eye. In the second, more realistic scenario, the object vergence is moved to about 0.9 m in front of the eye (O2), which corresponds to the retinal shift of 0.28 mm. In this approach, the axial length of the eye remains constant (I1). Figure 1 is a schematic illustration of the object and image planes in the described conditions.

In the first and second conditions, incident rays are defined by a pencil of collimated beams from an object at infinity. These rays are focused on the image plane which is located 18.57 mm and 18.29 mm behind the IOLs respectively. In the third condition, incident rays are defined by slightly diverging beams from an object converging to the image plane located 18.57 mm behind the IOLs. The rays are stimulated with a Gaussian distribution over the pupil in all of the conditions. In summary, condition (a) was shared by both IOLs and aimed to replicate their far-field imaging performance, while conditions (b) and (c) were designed for simulating and testing intermediate-distance imaging.

**Fig. 2:**
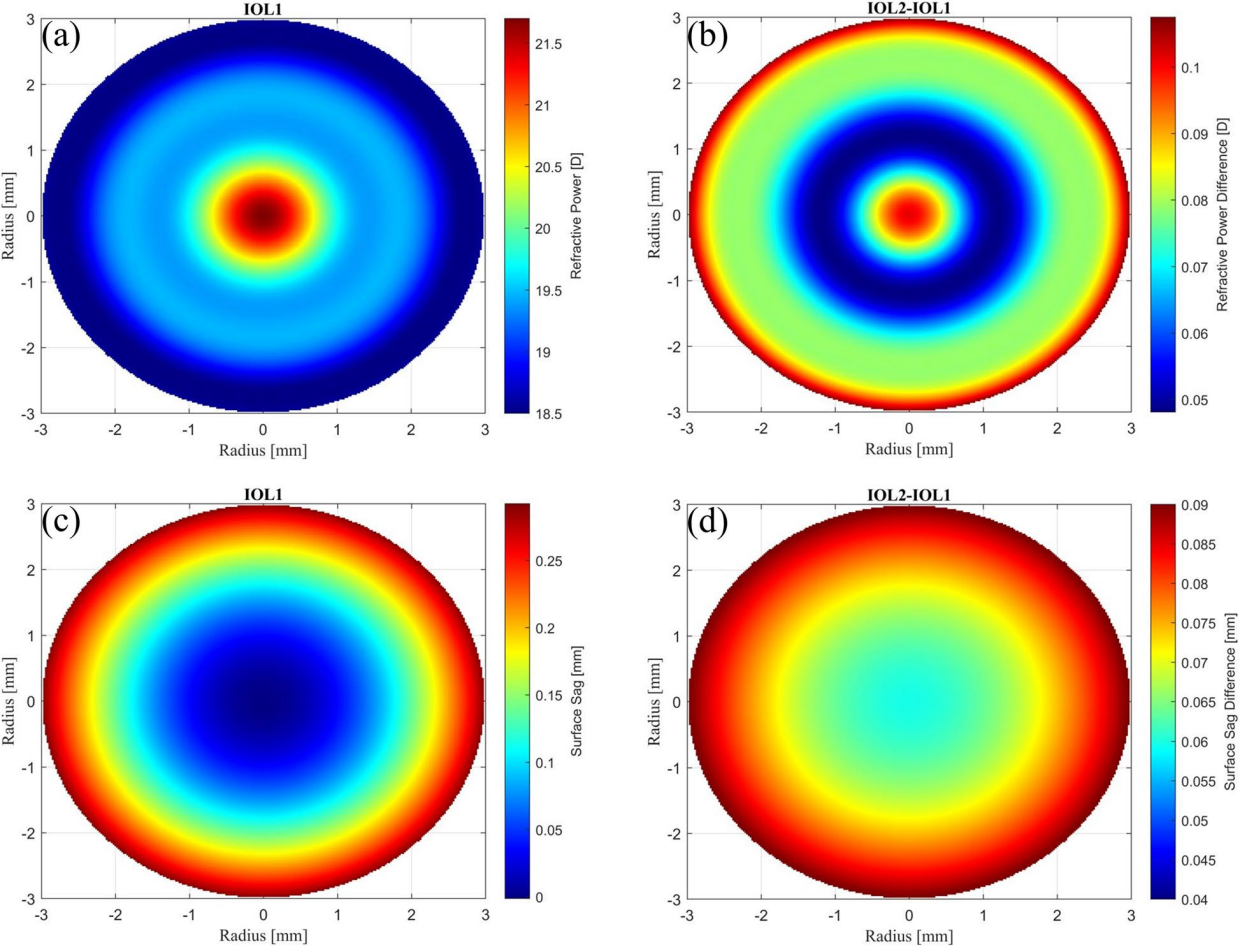
**a** Radial power profile of IOL1 optimized for scenario 1. **b** Subtracted refractive power of the IOL1 from the IOL2. **c** Surface sag profile of IOL1 optimized for scenario 1. **d** Subtracted surface sag profile of the IOL1 from the IOL2

Figure 2 illustrates the power profile and surface sag of IOL1, along with the differences in surface sag and power profiles between IOL1 and IOL2. These profiles are optimized for scenario 1 (shift in the image plane) and scenario 2 (shift in object vergence), respectively. This figure confirms that IOLs designed for the described scenarios exhibit slight differences in their surface and power profiles. Figure 2(b) reveals a maximum refractive power difference of 0.15 D in the peripheral region of the IOLs. In Fig. 2(d), the central zone of IOL2 exhibits minimal deviation in surface sag compared to IOL1, while the difference is most pronounced in the periphery, reaching up to 90 $\mu$m. Importantly, this figure exclusively portrays surface sag variations on the front surface of the IOLs, as their back surfaces are identical. Furthermore, it should be noted that the differences in surface characteristics can vary with different IOL powers, and consequently, IOLs with higher dioptric power may exhibit larger variances. However, even insubstantial surface variations can also influence the optical performance of the IOLs.

**Fig. 3:**
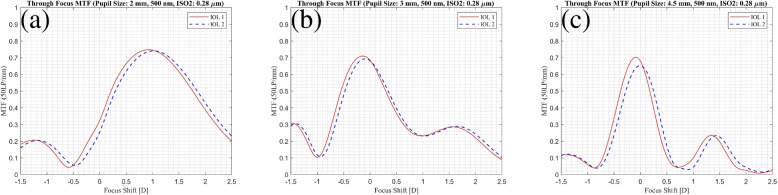
Through Frequency modulation transfer function (MTF) at 50 LP/mm for **a** 2 mm pupil diameter, **b** 3 mm pupil diameter, and **c** 4.5 mm pupil diameter. The solid and dashed lines represent IOL1 and IOL2 respectively

To provide a more precise comparison of optical performance, further evaluations are necessary. The MTF (Modulation Transfer Function) is a frequently employed method for contrasting various IOL designs, appraising clinical results, and gauging optical performance across diverse situations, including varying pupil sizes [11, 12]. Figure 3 presents the through-focus MTF at 50 LP/mm, assessed within an eye model that replicates the average corneal spherical aberration (0.28 $\mu$m) under green light (500 nm), across various pupil sizes for both IOL1 and IOL2. This figure demonstrates a variance in the position of the maximum MTF value between IOL1 and IOL2 across varying pupil sizes. The maximum MTF value consistently appears at a higher dioptric power for IOL2. However, the highest MTF values between the two IOLs do not exhibit a significant difference. It is known that MTF at a single spatial frequency is well correlated with contrast sensitivity measured clinically in pseudophakic patients [13, 14] but calculating the visual acuity requires multiple spatial frequencies [15]. However, the aim of the study is to utilize other metrics to employ a cross-validation approach to assess the imaging performance of each IOL under diverse conditions. IOL1 and IOL2 are tested in each of these three conditions (Fig. 1) separately. For each of them, we analyzed the Strehl ratio, the root-mean-square (rms) of the spot radius, and the spherical aberration term from a Zernike decomposition of the wavefront at the focal position. These analyses were conducted at a wavelength of 500 nm and with varying pupil diameters.

## Results

**Fig. 4:**
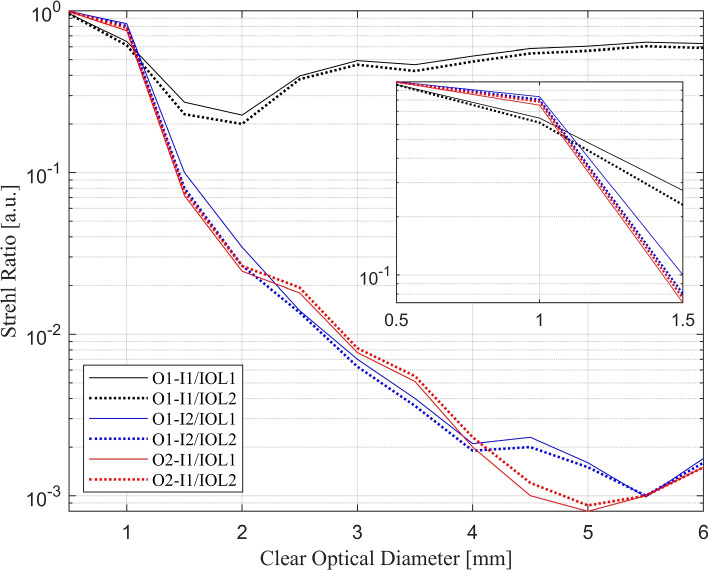
Strehl ratio of the focal spot as a function of pupil diameter. Strehl ratio is determined for I1/O1 (black), I2/O1 (blue), and I1/O2 (red). The solid and dotted lines represent IOL1 and IOL2 respectively

Figure 4 shows the Strehl ratio at the focal planes of two proposed IOLs in the presented test conditions as a function of clear optical diameter, which ranges from 0.5 to 6 mm. The vertical axis of Fig. 4 is shown on a logarithmic scale to make the difference between the obtained results more distinguishable. The box inside the figure illustrates the zoomed-in view of the graph in the small zone, which is allocated for intermediate vision.

In condition (a), the Strehl ratio varies from about 0.2 to 0.9 for both IOLs, and in 2 mm diameter results showed the lowest amount of Strehl ratio. In this condition, the outcomes are almost similar but IOL1 has a higher Strehl ratio than IOL2 in any pupil size. Unlike condition (a), in the two others, the Strehl ratio starts at about 0.99 for the small zone, which is higher than condition (a). However, the Strehl ratio decreases to 0.01 with increasing pupil diameter. The morphology of the first pair of curves (depicted in black) is influenced by the weighting coefficients assigned to various zones utilized within the optimization procedure. While the blue and red curves depict the inherent natural falloff for the near vision cases with an increase in pupil diameter.

**Fig. 5:**
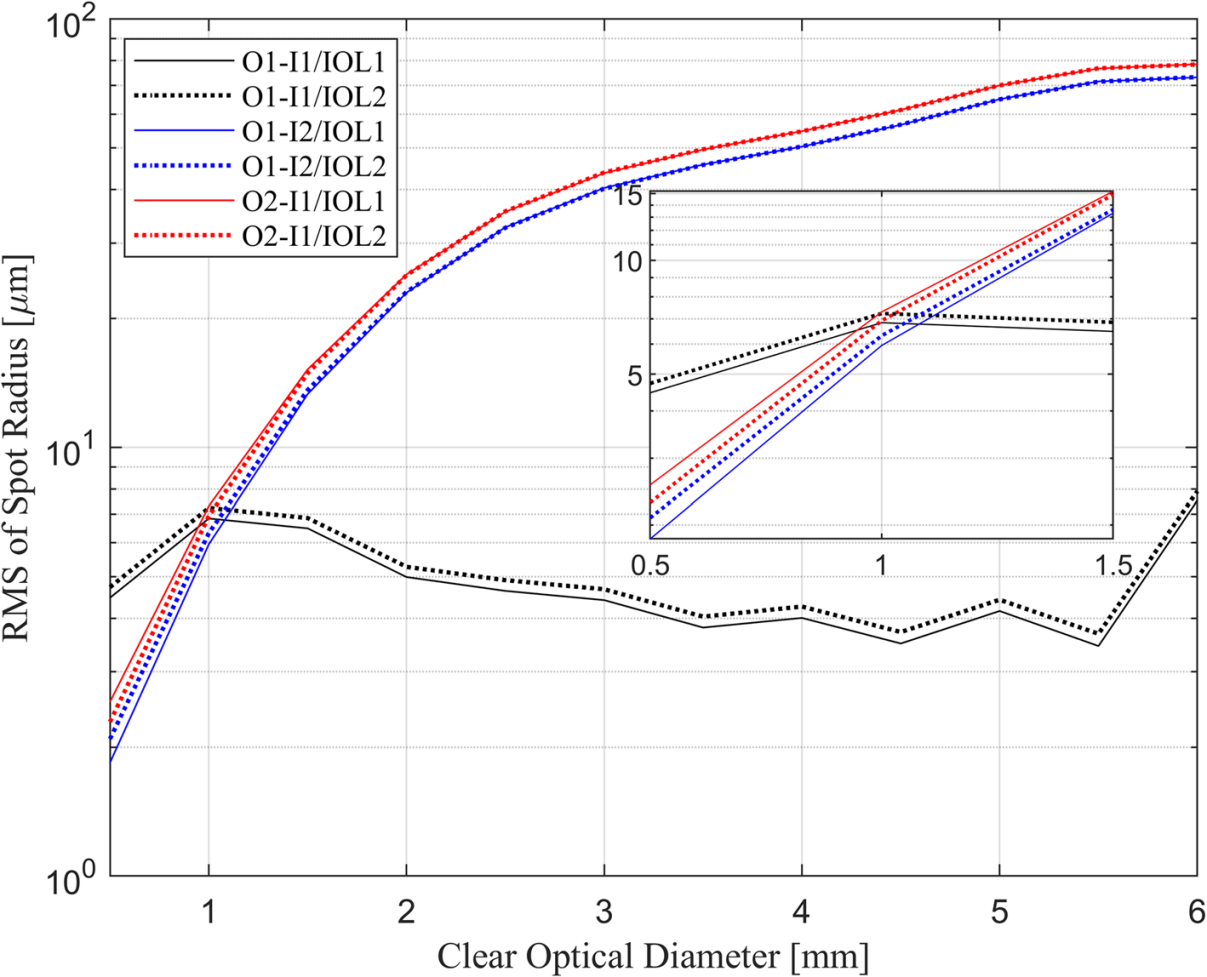
Root mean square (rms) of the ray scatter radius at the focal spot as a function of pupil diameter. The rms is determined for I1/O1 (black), I2/O1 (blue), and I1/O2 (red). The solid and dotted lines represent IOL1 and IOL2 respectively

Figure 5 shows the root mean square of the ray scatter radius at the focal planes of IOL1 and IOL2 in described conditions as a function of pupil diameter. The vertical axis of this figure is also shown on a logarithmic scale to make the difference between the obtained results more distinguishable. The box inside the figure illustrates the zoomed-in view of the graph in the small zone, which is allocated for intermediate vision.

In condition (a), the rms of the focal spot radii fluctuate between 3.5 $\upmu$m to 8 $\upmu$m. In conditions (b) and (c), the results do not exhibit significant differentiation and demonstrate a negligible difference. Like Fig. 4, the root-mean-square of spot radius behaviour is more similar in tests (b) and (c) in comparison to (a). In conditions (b) and (c), the spot size is smaller than in condition (a), but it is limited to a pupil diameter of 1 mm. For larger pupil diameters, the spot radii of the rays increase up to ten times larger than the maximum of condition (a) outcomes.

**Fig. 6:**
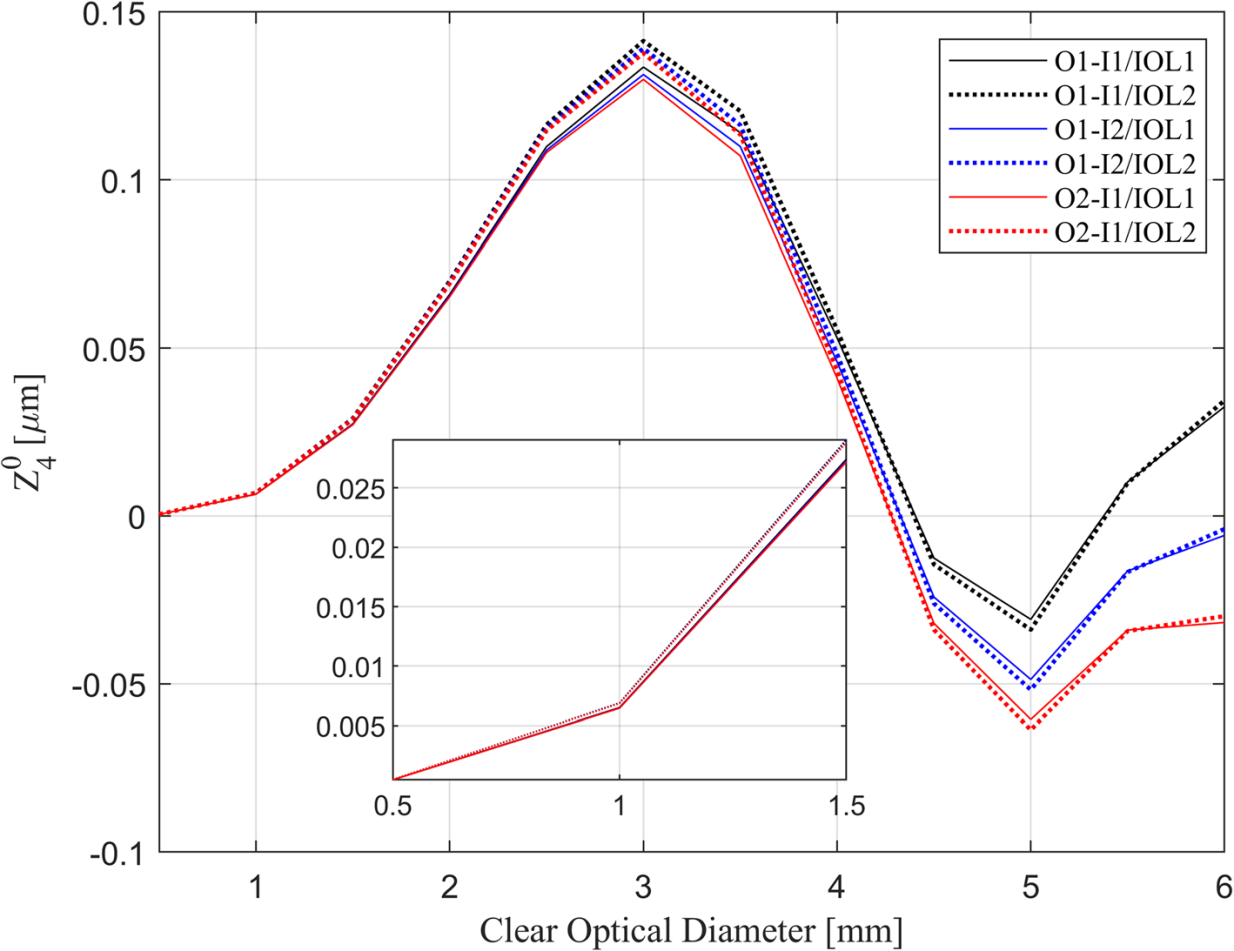
Spherical aberration of the wavefront as a function of pupil diameter. Spherical aberration is determined for I1/O1 (black), I2/O1 (blue), and I1/O2 (red). The solid and dotted lines represent IOL1 and IOL2 respectively

Finally, Zernike decomposition was performed to analyze the wavefront at the focal planes. Figure 6 shows the primary spherical aberration term (Z40) from the Zernike decomposition of the wavefront at the focal planes of both IOLs in 3 conditions as a function of pupil diameter.

As seen in the figure, in the small and medium zones (0-3 mm), the spherical aberration increases up to around 0.1 $\upmu$m with increasing pupil diameter. But for all test conditions, the minimum amount of spherical aberration is detected at 5 mm in diameter (around -0.04 $\upmu$m).

## Discussion

There is a lot of debate over the imaging performance of the different IOL types, especially multifocal and enhanced depth of focus lenses. After cataract surgery, the imaging performance of the pseudophakic eye is influenced by corneal topography (aberration), lens design, the alignment of the cornea and lens relative to the visual axis, and the shape of the pupil. The Liou-Brennan schematic model eye represents the average geometry of a human eye, and the behaviour of ocular imaging varies across different eyes. MF and EDOF lenses are designed to preserve pseudoaccommodation. To design MF or EDOF lenses for simultaneous imaging of far and near objects, the imaging properties of the cornea for both far and near-distance objects should be considered. Most studies concentrate on the cornea’s imaging properties for distant objects, ignoring the difference in imaging performance when viewing near objects.

In the present study, we designed two EDOF lenses, first without considering the imaging properties of the eye for near-distance objects (IOL1), and then with this consideration (IOL2). From the power profiles in Fig. 1, it is acceptable to categorize both as 20.0 + 1.5 D EDOF IOL. Extrapolating these profiles, first to the physical construction of each IOL and then to the potential clinical results, these IOLs could be indistinguishable under realistic conditions.

As expected, the IOLs showed roughly similar performance for far vision, since the arrangement of the image plane and object vergence (I1/O1) was identical for both. From Fig. 4, in the small zone, the Strehl ratio of IOL1 is relatively higher than that of IOL2 when the image plane is shifted (I2). However, when the object vergence is shifted (O2), the Strehl ratio for IOL1 is higher. The basic explanation is that IOL1 and IOL2 are optimized for retinal shift and object shift, respectively, and it is expected that they have better intermediate vision imaging performance in these test conditions. The same behaviour in the small zone is also observed in Fig. 5, where a lower rms value indicates relatively better IOL imaging capability for intermediate vision.

In the medium and large zones, on the other hand, the outcomes were more unpredictable. In these zones, results show that at different radii, each of the IOLs can have better imaging performance. That is because the medium zone is not directly optimized for the dominant intermediate vision, and the large zone was allocated only for far vision. However, with increasing radii, the imaging performance for near vision decreases significantly. Therefore, distant vision gradually becomes dominant. This implies that the poor intermediate vision imaging performance of the IOLs should be neglected in these zones, regardless of the optimization scenario. In Fig. 6, the spherical aberration of the IOLs in the small zone is approximately equal. With increasing diameter, the detected spherical aberrations of IOL1 and IOL2 diverge and become more distinguishable. However, IOL1 exhibits higher spherical aberration than IOL2, but only up to a pupil size of 4 mm. Beyond this threshold, the difference diminishes to imperceptible levels, and at 5 mm, an opposite effect can be observed. This figure also demonstrates that within the larger zone, the difference in spherical aberration of each condition is more substantial. As described before, adding refractive power to the central zone requires surface geometry modification. These modifications increase or decrease the curvature of the surface. As a result, the spherical aberration varies in different zones. The inequality of the corneal spherical aberration for near and far objects [16] causes a slight geometrical difference between the front surfaces of the IOLs. In conclusion, the imaging performance of the EDOF IOLs is affected by object distance in the design scenarios. Although this dissimilarity between the imaging performance of the two IOLs is not significant, it is better to take it into account to have more realistic simulations and test scenarios.

### Limitations

This study has the character of a pilot study and our principal aim has been to inform new IOL designers about IOL optimization strategy. The results cannot prove that optimization with object shift is superior to image plane shift, and there are no clinical outcomes to support this claim, even though there is a slight difference between the two optimization strategies. Therefore, more data are required to confirm any improvement in the imaging performance of IOLs designed with a more realistic scenario. Moreover, IOL manufacturers do not present references confirming the common design criteria for EDOF IOLs. But the authors made an attempt to investigate the effect of object and image lane variances in computing.

## Conclusion

Generally, the fact that imaging performance is affected by object distance is true for any optical system, and the human eye is no exception to this principle. Our simulation study recommends considering the difference between object shift and image plane shift, especially when designing multifocal or enhanced depth of focus lenses to achieve more accurate pseudoaccommodation after cataract surgery.

### Supplementary Information

**Additional file 1.**

## Data Availability

The authors confirm that the data supporting the findings of this study are available within the article.
